# Fat content, fatty acid pattern and iron content in livers of turkeys with hepatic lipidosis

**DOI:** 10.1186/s12944-017-0484-8

**Published:** 2017-05-30

**Authors:** Christian Visscher, Lea Middendorf, Ronald Günther, Alexandra Engels, Christof Leibfacher, Henrik Möhle, Kristian Düngelhoef, Stefan Weier, Wolfram Haider, Dimitri Radko

**Affiliations:** 1 Institute for Animal Nutrition, University of Veterinary Medicine Hannover, Foundation, Bischofsholer Damm 15, D-30173 Hannover, Germany; 2 Heidemark GmbH, Veterinärlabor, Jakob-Uffrecht-Str. 20, D-39340 Haldensleben, Germany; 3 Tierarztpraxis Dr. A. Engels, Gut Hacheney 2-5, D-59199 Bönen-Lenningsen, Germany; 4 Tierärztliche Gemeinschaftspraxis Dres. Windhaus & Hemme, An der Ohe 1, D-49377 Vechta, Germany; 5 Tierarztpraxis an der Güterstraße, Güterstraße 7, D-46499 Hamminkeln, Germany; 6 Praxis am Bergweg, Bergweg 20, D-49393 Lohne (Oldenburg), Germany; 7 Institut für Tierpathologie, Schönhauser Str. 62, D-13127 Berlin, Germany; 8 Elanco Animal Health GmbH, Werner-Reimers-Str. 2-4, Bad Homburg, D-61352 Germany

**Keywords:** Hepatic lipidosis, Hepatic fatty acid pattern, Iron, Steatohepatitis

## Abstract

**Background:**

The so-called “hepatic lipidosis” in turkeys is an acute progressive disease associated with a high mortality rate in a very short time. Dead animals show a massive fatty degeneration of the liver. The cause is still unclear. Previous findings suggest that there may be parallels to human non-alcoholic fatty liver disease. The object of the study was to examine the changes in the fat contents, the fatty acid composition and the iron content in livers of animals, which have died from hepatic lipidosis.

**Methods:**

The conspicuous livers (*n* = 85) were collected from 20 flocks where the phenomenon of massive increased animal losses accompanied by marked macroscopically visible pathological liver steatosis suddenly occurred. For comparison and as a reference, livers (*n* = 16) of two healthy flocks were taken. Healthy and diseased flocks were fed identical diets concerning official nutrient recommendations and were operating under standardized, comparable conventional conditions.

**Results:**

Compared to livers of healthy animals, in the livers of turkeys died from hepatic lipidosis there were found massively increased fat levels (130 ± 33.2 vs. 324 ± 101 g/kg dry matter-DM). In all fatty livers, different fatty acids concentrations were present in significantly increased concentrations compared to controls (palmitic acid: 104 g/kg DM, +345%; palmitoleic acid: 18.0 g/kg DM, + 570%; oleic acid: 115 g/kg DM, +437%). Fatty acids concentrations relevant for liver metabolism and inflammation were significantly reduced (arachidonic acid: 2.92 g/kg DM, −66.6%; eicosapentaenoic acid: 0.141 g/kg DM, −78.3%; docosahexaenoic acid: 0.227 g/kg DM, −90.4%). The ratio of certain fatty acids to one another between control and case livers changed analogously to liver diseases in humans (e.g.: C18:0/C16:0 – 0.913 against 0.311; C16:1n7/C16:0 – 0.090 against 0.165; C18:1/C18:0 – 0.938 against 4.03). The iron content in the liver tissue also increased massively (271 ± 51.5 vs 712 ± 214 mg/kg DM).

**Conclusion:**

The hepatic lipidosis has a massive impact on the lipid content, the lipid composition and the iron content in the liver. The character of the metabolic disorder includes parallels to the non-alcoholic steatohepatitis in humans.

## Background

On turkey farms, a devastating disease associated with massive changes in hepatic tissue has already been described in literature for more than 20 years [1]. The disease is associated with sudden death in up to 15% of animals in flocks without previous clinical signs of illness [1, 2]. In practice, a certain impaired blood coagulation is also described. The typical pathological changes in dead animals focus on the liver [2]. Fat accumulation in hepatocytes, leading to the formation of large vacuoles in the cytoplasm, fatty degeneration, severe and multifocal, acute, hemorrhagic, necrotising hepatitis with eosinophilic intranuclear inclusions are typical signs in animals which died from hepatic lipidosis [2, 3]. In severe cases signet ring cells occur [4]. The etiology and pathogenesis is still unclear. Both metabolic and infectious causes are discussed [1, 2]. In human medicine a disease has been occurring for years with similar pathological changes. The nonalcoholic fatty liver disease (NAFLD) in the harmless form as simple steatosis (SS) or in the more serious variant as nonalcoholic steatohepatitis (NASH) is increasingly recognized as the hepatic manifestation of insulin resistance and the systemic complex known as metabolic syndrome [5].

Unlike in humans, where a standardized and validated system for the histological evaluation of the fatty liver disease exists [5], these methods are missing in poultry. Nevertheless, to gain a better understanding of the pathogenesis of the so-called “hepatic lipidosis” in turkeys the fatty acid composition is of interest. The ratios of destinct fatty acids in the liver are correlated to the steatosis score in humans, others are associated with the lobular inflammation score [6]. Additionally, the iron content in liver samples is important in order to be able to assess the involvement of infections or kind of metabolic disorders [7].

The object of the study was to examine the changes in the fat contents, the fatty acid composition and the iron content in livers of animals, which have died from hepatic lipidosis to get a step closer to the triggers of this disease.

## Methods

The study took place in cooperation with German poultry veterinarians. Only veterinarians have been addressed who had already observed the phenomenon of hepatic lipidosis in turkeys during the previous years. Therefore, these persons were optimally trained in clinical diagnosis. Only samples from clinically apparent cases were obtained when it was ensured that the conditions of feeding and the diets corresponded to the conventional standard. Thus, the conditions were comparable between all farms and did not differ from non-affected flocks. In case of a hepatic lipidosis in fattening turkey flocks, livers were collected from four to five dead animals and used for further investigations. Sample collection was done in the pathology unit of the specific veterinary practices. So this study was not based on an animal experiment requiring a notification or an approval according to the Animal Protection Act. Interventions were carried out only on dead or slaughtered animals.

From January 2015 to April 2016, a total of 85 samples were collected from 20 different diseased flocks. For comparison, samples (*n* = 16) from healthy slaughtered turkeys from two non-affected flocks were collected.

### Animals and feeding

In the investigations exclusively commercial turkeys from line “B.U.T. Big 6” were included. In eighteen out of twenty affected flocks, only female animals were kept and in two only male turkeys. The farm size differed between 10,000–30,000 animals each. Animals were fed a commercial complete diet. The feeding programme consisted as usual of several phases, which were adapted to the energy and nutrients demands with regard to the age of the birds (Table 1). The individual farms were supplied by several feed mills. In the majority of cases, the phenomenon occurred during the fattening period at the time of the fourth and fifth feeding phase. These diets no longer contained coccidiostats. All animals were fed ad libitum.

**Table 1:** Nutrient composition of the case diets at time of outbreak of hepatic lipidosis in flocks

Item	Nutrient content (g/kg diet)^a^		Item	Nutrient content (g/kg diet)	
	Mean	SD		Mean	SD
ME (MJ/kg diet)	12.4	0.20	Arginine	11.0	1.39
			Cysteine	2.94	0.35
Crude ash	45.8	7.58	Isoleucine	6.89	0.76
Crude fat	64.0	7.10	Leucine	13.7	1.41
Crude fibre	29.1	3.65	Lysine	11.4	0.95
Crude protein	177	15.0	Methionine^b^	3.39	1.16
Starch	414	15.6	Phenylalanine	8.36	1.15
Sugar	37.9	4.27	Threonine	6.56	0.97
Palmitic acid	13.8	6.79	Valine	8.12	0.70
Palmitoleic acid	0.11	0.02	Alanine	7.85	0.81
Margaric acid	0.06	0.01	Aspartic acid	13.6	2.56
Stearic acid	1.84	0.49	Glutamic acid	36.5	2.35
Oleic acid	18.7	2.47	Glycine	7.56	0.62
Linoleic acid	23.5	1.58	Histidin	4.58	0.36
α-Linolenic acid	1.67	0.19	Proline	12.6	0.74
Arachidic acid	0.24	0.03	Serine	8.93	1.07
Iron (mg/kg diet)	226	42.8	Tyrosine	6.16	0.75

Nutrient content = amounts of raw nutrients, fatty acids, iron and amino acids

^a^In total eight diets were analysed; ^b^Only DL-methionine

### Sample collection

**Fig. 1:**
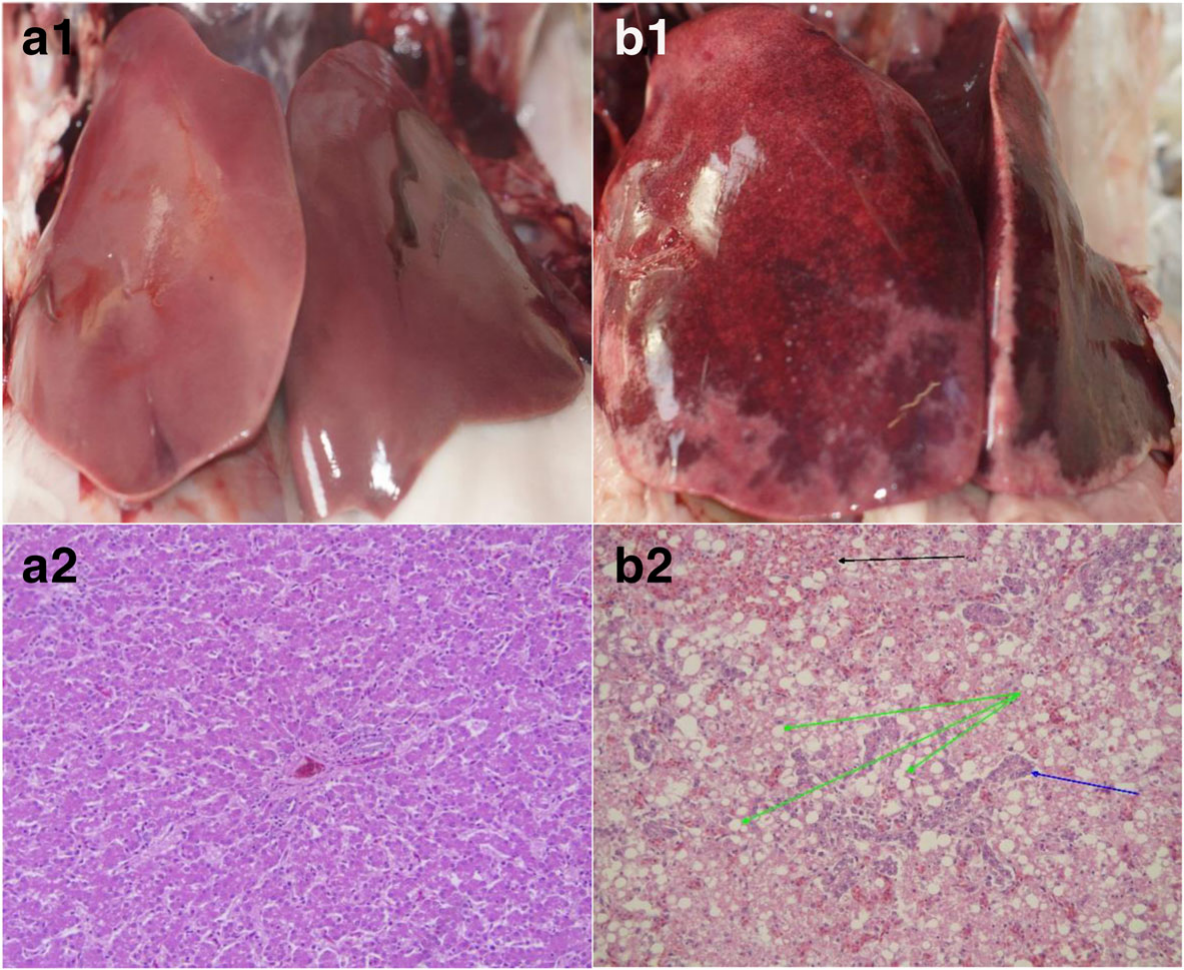
Typical macroscopic image of livers from healthy turkeys (*a1*) and deceased turkeys with hepatic lipidosis (*b1*) and histological pictures of livers from healthy turkeys (*a2*, HE-staining, ×20) and deceased turkeys with hepatic lipidosis (*b2*, HE-staining, ×20; *black* arrow: dilated sinusoids filled with blood; *blue* arrow: biliary duct proliferates; *green* arrows: liver cells with fat vacuoles)

At the time of illness the animals were on average about 90–95 days of age. The extent of losses due to the illness varied. Between 1.5% and 10% total losses were due to hepatic lipidosis. Within a few hours four to five animals that had died suddenly were taken randomly and transferred immediately to pathology of each veterinary practice. Here the removal of the liver took place under sterile conditions. A systematic macroscopic assessment as in humans [5] was not possible because in each practice different investigators took the samples. Whole liver samples were taken (Fig. 1, b1), stored in plastic screw vials (500 mL, Sarstedt, Germany) and sent on ice immediately to the laboratory.

### Chemical analysis

Samples of all diets were analysed by standardized laboratory methods according to the VDLUFA [8]. The analyses were always performed in duplicate. The dry matter content was determined by drying to the weight constancy at 103 °C. The crude ash was analysed by means of incineration in the muffle furnace at 600 °C for 6 h. The total nitrogen content was determined by means of the analyser Vario Max® (Elementar, Hanau, Germany), which operates according to the principle of a catalytic tube combustion (DUMAS combustion method). The molecular nitrogen formed by reduction from nitric oxide was detected by a thermal conductivity detector and the nitrogen content was calculated by the device software. The crude protein content of the sample was calculated by multiplication with a constant factor of 6.25. The dietary content of crude fibre was determined after washing in diluted acids and alkalis by established methods. The determination of starch contents was carried out polarimetrically (Polatronic E, Schmidt und Haensch GmbH & Co., Berlin, Germany). The sugar content was analysed according to Luff-Schoorl by titration with sodium thiosulphate. Amino acids in feed samples were determined by ion-exchange chromatography (AA analyser LC 3000, Biotronic, Maintal, Germany). Results were evaluated according to established methods [9]. In feed and liver samples the crude fat content was determined after acid hydrolysis in the Soxhlet apparatus. For the calculation of nutrient concentration in the fat-free DM, the absolute fat content was subtracted from the DM content of a sample. The mineral content was analysed in accordance with the official methods [8] by atomic absorption spectrometry (Unicam Solaar 116, from Thermo, Dreieich, Germany). The determination of levels of long chain fatty acids in liver samples was carried out using established methods [10]. Exactly 200 mg of liver tissue was placed in a glass tube. A methanol-hexane-tridecanoic-acid mixture was utilised as standard. Subsequently, acetyl chloride was added and the sample was heated, followed by the addition of potassium chloride solution. The measurement was carried out by gaschromatography (GC TRACE 1300, ThermoScientific®, Dreieich, Germany; SP-2560 Column, Supelco, Bellefonte, USA; carrier gas: nitrogen) after centrifugation with the superior hexane phase.

### Statistical analysis

The statistical analysis of the data was performed using the Statistical Analysis System for Windows, the SAS® Enterprise Guide®, version 7.1 (SAS Institute Inc. Cary, USA). First, for statistical analysis normal distribution of data was verified. For that residuals of the data of single parameters were compared with the normal distribution. After that one-way ANOVA was calculated in the presence of a normal distribution (Ryan Einot-Gabriel-Welsch statistics). In non-normally distributed data, the Wilcoxon test was carried out.

For the purpose of evaluating parameters like iron content and certain fatty acids concentrations in liver, fatty acid classes (fl) for liver tissue were defined according to the total amount of long chain fatty acids analysed in dry matter (DM) of liver tissue: fl 1: ≤ 200 g/kg DM; fl 2: > 200–250 g/kg DM; fl 3: > 250–300 g/kg DM; fl 4: > 300–350 g/kg DM; fl 5: > 350–400 g/kg DM; fl 6: > 400–450 g/kg DM; fl 7: > 450 g/kg DM. This was followed by a comparative analysis of each parameter with one-way ANOVA (Ryan Einot-Gabriel-Welsch statistics).

For the correlation analysis of normal distributed data the correlation coefficient of Pearson was used. In non-normally distributed residuals the rank correlation coefficient according to Spearman was calculated. All statistical tests were two-sided; a *P*-value <0.05 was considered significant.

## Results

Until there was an outbreak of the disease, the animal losses on the farms amounted to normal values. The complete diets used on the farms completely matched usual turkey diets. Performance level in flocks was high up to the outbreak of illness and exceeded the performance goals of the genetic line.

### Fat content and fatty acid composition of liver tissue

To compare the liver composition of healthy and affected animals, a total of 101 liver samples from 22 farms were tested. These were 16 livers from healthy animals and 85 livers from affected animals from flocks suffering from hepatic lipidosis. The fat content in the liver of affected animals was approximately three times higher than the content in the livers of healthy birds, thus significantly increased (control: 123 ± 36.6; case: 345 ± 103 g/kg DM). The sum of the individual fatty acids changed in an identical manner (control: 130 ± 33.2; case: 324 ± 101 g/kg DM).

With regard to the absolute content of the individual fatty acids significant differences between the levels in control livers and case-livers were seen. Concerning long chain fatty acids (Table 2) significant differences were only missing for stearic acid.

**Table 2:** Comparative overview of the content, the relative changes in the fatty acid concentration in the liver

Item^c^	Content		Relation^d^	Relative share^e^	
	[g/kg liver DM ± SD]		[% of control]	[% ± SD]	
	Control	Case	Case	Control	Case
Myristic acid C14:0	0.411^b^ ± 0.138	1.93^a^ ± 0.936	469 ± 228	0.328^b^ ± 0.124	0.582^a^ ± 0.192
Trans-Myristic acid C14:1	0.070^b^ ± 0.070	0.350^a^ ± 0.208	504 ± 299	0.045^b^ ± 0.040	0.103^a^ ± 0.050
Palmitic acid C16:0	30.2^b^ ± 8.41	104^a^ ± 32.6	345 ± 108	23.3^b^ ± 1.29	32.1^a^ ± 3.09
Palmitoleic acid C16:1	3.17^b^ ± 2.80	18.0^a^ ± 9.62	570 ± 304	2.08^b^ ± 1.67	5.27^a^ ± 2.01
Margaric acid C17:0	0.244^b^ ± 0.103	0.495^a^ ± 0.208	202 ± 85.2	0.215^a^ ± 0.139	0.169^a^ ± 0.135
Stearic acid C18:0	26.5^a^ ± 3.22	28.3^a^ ± 5.47	107 ± 20.7	21.1^a^ ± 3.13	9.79^b^ ± 4.13
Elaidic acid C18:1n9t	0.395^b^ ± 0.199	1.27^a^ ± 0.516	320 ± 131	0.287^b^ ± 0.109	0.389^a^ ± 0.123
Oleic acid C18:1n9c	26.4^b^ ± 18.8	115^a^ ± 43.9	437 ± 166	18.1^b^ ± 10.1	34.3^a^ ± 7.02
Linoleic acid C18:2n6c	29.3^b^ ± 3.54	45.9^a^ ± 20.9	157 ± 71.2	23.5^a^ ± 4.26	14.4^b^ ± 4.55
α-Linolenic acid C18:3n3	0.547^b^ ± 0.180	2.61^a^ ± 1.68	478 ± 307	0.418^b^ ± 0.068	0.755^a^ ± 0.387
Gadoleic acid C20:1	0.294^b^ ± 0.112	0.624^a^ ± 0.298	212 ± 101	0.220^a^ ± 0.047	0.193^a^ ± 0.081
Arachidonic acid C20:4n6	8.75^a^ ± 1.67	2.92^b^ ± 2.08	33.4 ± 23.8	7.17^a^ ± 2.48	1.37^b^ ± 2.62
Eicosapentaenoic acid C20:5n3	0.651^a^ ± 0.449	0.141^b^ ± 0.122	21.7 ± 18.8	0.566^a^ ± 0.512	0.048^b^ ± 0.053
DHA Docosahexaenoic acid C22:6n3	2.37^a^ ± 2.96	0.227^b^ ± 0.254	9.59 ± 10.8	2.33^a^ ± 3.15	0.120^b^ ± 0.342

^c^Medium and long chain fatty acids in concentrations >0.5 g/kg DM were considered in the comparison

^d^In animals with hepatic lipidosis in relation to the fatty acid concentrations in the livers of healthy animals (basis for calculation = 100%)

^e^Percentage distribution of medium and long chain fatty acids (FA) in liver tissue of healthy (*n* = 16) and deceased (*n* = 85) animals from conspicuous flocks with hepatic lipidosis

^a,b^Values within a row concerning object of investigation with different superscripts differ significantly at *P* < 0.05

The absolute contents of arachidonic acid, eicosapentaenoic acid and docosahexaenoic acid were significantly lower in livers of animals that died from hepatic lipidosis. The content of other fatty acids was higher in affected animals. With respect to the relative proportions of the corresponding fatty acids significant shifts were revealed. While the relative proportion of margaric acid and gadoleic acid remained unchanged, the amount of stearic acid, linoleic acid, arachidonic acid, eicosapentaenoic acid and docosahexaenoic acid, however, was significantly lower. The share of other fatty acids increased.

The comparison at fatty acid category level (Table 3) shows for the fatty acids with the highest proportions in the liver a continuous increase in saturated and monounsaturated fatty acids at higher fat content. In the case of palmitic acid, palmitoleic acid and oleic acid, it was significantly higher in this category. For linoleic acid and α-linolenic acid the second highest category of fat was associated with the highest fatty acid content. In the highest category, fatty acid values were significantly smaller again.

**Table 3:** Long chain fatty acids and fatty acid ratios related to metabolism pathways [1] in liver tissue

Item		Control (n = 16)		Case													
Acid	[]			fl1 (*n* = 8)		fl2 (*n* = 10)		fl3 (*n* = 13)		fl4 (*n* = 10)		fl5 (*n* = 17)		fl6 (*n* = 12)		fl7 (*n* = 9)	
		Mean	SD	Mean	SD	Mean	SD	Mean	SD	Mean	SD	Mean	SD	Mean	SD	Mean	SD
Palmitic	g/kg DM	30.2^e^	8.41	36.1^e^	21.5	77.6^d^	8.39	90.9^c^	6.33	102^c^	8.98	123^b^	12.2	128^b^	10.8	151^a^	9.40
Palmitoleic		3.17^c^	2.80	5.06^c^	5.01	8.60^c^	3.35	16.6^b^	3.55	16.3^b^	4.74	21.0^b^	6.47	22.5^b^	8.08	33.6^a^	9.24
Stearic		26.5^cd^	3.22	20.0^e^	2.84	25.6^d^	3.08	26.1^cd^	3.02	28.2^bcd^	3.82	30.7^abc^	4.89	31.6^ab^	4.84	33.3^a^	6.14
Oleic		26.4^e^	18.8	27.1^e^	24.6	67.7^d^	11.2	104^c^	12.2	117^c^	14.5	136^b^	13.4	146^b^	17.9	179^a^	19.6
Linoleic		29.3^e^	3.54	16.4^f^	3.50	33.0^de^	8.12	32.8^de^	11.0	45.3^cd^	12.2	50.1^bc^	14.4	75.1^a^	13.9	59.7^b^	21.3
α-linoleic		0.55^d^	0.18	0.28^d^	0.17	1.78c	0.73	1.60c	0.95	2.67bc	1.08	2.62bc	1.08	5.13a	1.31	3.58b	1.47
Arachidonic	g/kg DM	8.75^a^	1.67	5.52^b^	4.75	2.89^c^	1.76	2.33^c^	1.37	2.99^c^	1.29	2.17^c^	1.33	3.24^c^	1.14	2.35^c^	1.36
Eicosapentaenoic		0.65^a^	0.45	0.11^b^	0.12	0.10^b^	0.06	0.09^b^	0.09	0.14^b^	0.08	0.15^b^	0.15	0.20^b^	0.13	0.20^b^	0.18
Docosahexanoic		2.37^a^	2.96	0.59^b^	0.67	0.19^b^	0.11	0.12^b^	0.06	0.16^b^	0.10	0.21^b^	0.11	0.23^b^	0.16	0.25^b^	0.19
Ratio																	
C18:0/C16:0 [1]	g/kg DM	0.91^a^	0.15	0.71^b^	0.31	0.33^c^	0.04	0.29^c^	0.04	0.28^c^	0.04	0.25^c^	0.04	0.25^c^	0.04	0.22^c^	0.03
C16:1n7/C16:0 [1]		0.09^c^	0.07	0.11^bc^	0.10	0.11^bc^	0.04	0.18^ab^	0.04	0.16^ab^	0.05	0.17^ab^	0.06	0.17^ab^	0.06	0.22^a^	0.07
C18:1n9/C16:0 [1]		0.94^d^	0.62	1.32^d^	1.17	2.69^c^	0.63	4.06^b^	0.67	4.20^b^	0.62	4.57^ab^	1.08	4.74^ab^	1.07	5.59^a^	1.36
n-6/n-3 [1]		23.5^ab^	12.5	30.3^a^	9.68	20.0^b^	5.90	23.8^ab^	10.3	18.4^b^	4.66	19.3^b^	4.22	15.0^b^	1.75	17.0^b^	4.01

Fat level 1 = fl 1: ≤200 g/kg DM; Fat level 2 = fl 2: >200–250 g/kg DM; Fat level 3 = fl 3: >250–300 g/kg DM; Fat level 4 = fl 4: > 300–350 g/kg DM; Fat level 5 = fl 5: > 350–400 g/kg DM; Fat level 6 = fl 6:> 400–450 g/kg DM; Fat level 7 = fl 7: > 450 g/kg DM

^a,b,c,d,e^ Values within a row with different superscripts differ significantly at *P* < 0.05

The contents of the n-6 fatty acid arachidonic acid and the n-3 fatty acids eicosapentaenoic acid and docosahexaenoic acid were significantly lower in the livers of animals with hepatic lipidosis (Table 3) but within the different fat level categories there were no differences in livers of diseased animals for the eicosapentaenoic acid and docosahexaenoic acid. The content of arachidonic acid decreased gradually. Here, the category fl 1 had, nevertheless, still significantly higher arachidonic acid content than the following fatty acid categories.

The ratio of C18:1 to C18:0, equivalent to the activity of Δ 9 desaturase, was significantly increased in the livers of affected animals (Table 4). Both, the n-6 - and n-3 pathway indicated significantly lower values in the case group. The ratio of n-6 to n-3 fatty acids according to [11] was not significantly different. The ratio of stearic acid to palmitic acid as well as the ratio of palmitoleic acid to palmitic acid and oleic acid to palmitic acid were significantly lower in affected animals. The ratio n-6 to n-3 according [6] showed no significant differences.

**Table 4:** Comparative overview of the different fatty acid ratios according to different authors [6, 11]

Ratio	Control (*n* = 16) Mean ± SD	Case (*n* = 85) Mean ± SD
“∆9 desaturase” [11] (C18:1/C18:0)	0.938^b^ ± 0.616	4.03^a^ ± 1.47
“n6- pathway” [11] (C20:4/C18:2)	0.300^a^ ± 0.056	0.082^b^ ± 0.118
“n3- pathway” [11] (C20:5 + C22:6/C18:3)	7.99^a^ ± 11.4	0.692^b^ ± 3.26
Ratio n6/n3 [11] (C18:2 + C20:2 + C20:3 + C20:4/C18:3 + C20:5 + C22:6)	17.3^a^ ± 8.34	18.8^a^ ± 6.25
Ratio C18:0/C16:0 [6]	0.913^a^ ± 0.152	0.311^b^ ± 0.165
Ratio C16:1n7/C16:0 [6]	0.090^b^ ± 0.071	0.165^a^ ± 0.066
Ratio C18:1n9/C16:0 [6]	0.938^b^ ± 0.616	4.03^a^ ± 1.47
Ratio n6/n3 [6] (C18:2n6 + C20:3n6 + C20:4n6/C18:3n3 + C22:6n3)	23.5^a^ ± 12.5	20.1^a^ ± 7.30

^a,b^Values within a row concerning object of investigation with different superscripts differ significantly at *P* < 0.05

The comparison of fatty acid ratios at fat category level (Table 3) shows that the ratio of C18:0 to C16:0 was significantly lower in the liver of animals that had died from hepatic lipidosis. In deceased animals, livers with a fat content greater than 200 g again had a significantly lower ratio. The ratio of C16:1n7 to C16:0 showed an opposite trend. In the control group, the ratio was 0.09, and thus significantly lower than in deceased animals. The significantly highest ratio was found in animals with a liver fat content of >450 g (Table 3). Here the ratio was 0.22.

The ratio of C18:1n9 to C16:0 was significantly lower in livers of healthy animals as well as in affected animals whose livers had a fat content of less than 200 g (c: 0.938 ± 0.616; fl 1: 1.33 ± 1.17). In relation to that, dead animals with higher fat content in the liver had significantly higher ratios (fl 2: 2.69 ± 0.634; fl 3: 4.06 ± 0.672; fl 4: 4.20 ± 0.621; fl 5: 4.57 ± 1.08; fl 6: 4.74 ± 1.07; fl 7: 5.59 ± 1.36).

The ratio of n-6 to n-3 fatty acids was significantly highest in the group of case animals and simultaneously still had a liver fat content of <200 g (fl 1: 30.3 ± 9.68) compared to the levels in livers of other fatty acid categories. In relation to the healthy animals, there were no significant differences (c: 23.5 ± 12.5).

There was a very strong Spearman rank correlation coefficient between the sum of the fatty acids in the liver tissue and the levels of palmitic acid (0.95) and the content of oleic acid (0.93, Table 5). There was also a very strong correlation coefficient between trans-myristic acid and palmitoleic acid, palmitic acid and oleic acid, palmitoleic acid and oleic acid and linoleic acid and alpha-linolenic acid, respectively.

**Table 5:**
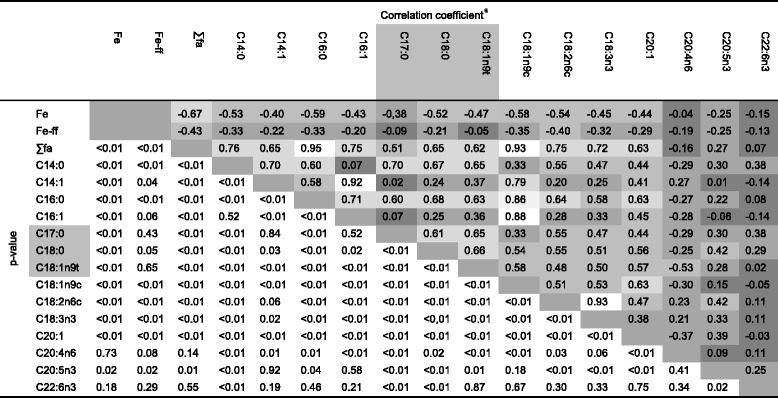
Crosstab regarding intercorrelations between iron content, total fatty acids and individual fatty acids in the liver tissue of deceased animals

^a^Pearson correlation coefficients – for combination of fatty acids between grey boxes; Spearman correlation coefficients – combination of colourless boxes); correlations: 00-.19 “very weak”; .20-.39 “weak”; .40-.59 “moderate”; .60-.79 “strong”; .80-1.0 “very strong”

A strong negative correlation was found between the level of fatty acids in the liver and the iron content, the contents of myristic acid, trans-myristic acid, palmitoleic acid, stearic acid, oleic acid, linoleic acid, alpha-linolenic acid and gadoleic acid in the liver. The contents of myristic acid and trans-myristic acid, palmitic acid, margaric acid, stearic acid, elaidic acid, linoleic acid and gadoleic strongly correlated as did the contents of trans-myristic acid and oleic acid. The levels of palmitic acid and palmitoleic acid, margarinic, stearic acid, oleic acid, linoleic acid, and gadoleic acid also highly correlated as did oleic acid and gadoleic acid. There existed a strong Pearson correlation between heptadecenoic acid and stearic acid, heptadecenoic acid and elaidic acid and between stearic and elaidic acid (Table 5).

### Iron content in the liver tissue

The iron content in the liver tissue also increased massively (control: 271 ± 51.5; case: 712 ± 214 g/kg DM) as well as the iron content in fat free liver tissue (control: 311 ± 64.7; case: 1084 ± 249 g/kg DM). Therefore, calculated on the fat-free basis it was more than three times higher.

**Fig. 2:**
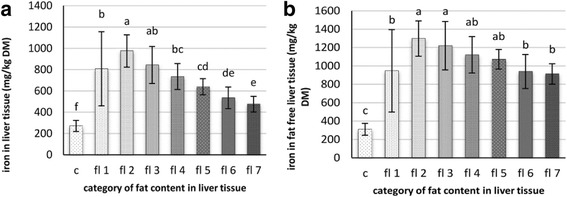
Mean levels of iron in the liver tissue (**a**) and iron in the fat free liver tissue (**b**) as a function of group membership (control [c, *n* = 16] or case [fl1–fl7, *n* = 85]) or the sum of fatty acids content in the liver (fl 1: ≤200 g/kg DM, *n* = 8; fl 2: >200–250 g/kg DM, *n* = 10; fl 3: >250–300 g/kg DM, *n* = 13; fl 4: > 300–350 g/kg DM, *n* = 10; fl 5: > 350–400 g/kg DM, *n* = 17; fl 6:> 400–450 g/kg DM, *n* = 12; fl 7:> 450 g/kg DM, *n* = 9); *p* < 0.05

The statistical analysis of the iron content versus fat classes in the liver tissue showed that the iron content was significantly higher in the category >200–250 g fat/kg DM liver tissue (Fig. 2). With increasing fat content in the liver, the iron contents in liver tissue were lower. When calculating the levels of iron in the liver to the fat-free mass of liver issue, the iron content in absolute terms was higher. The absolute differences, however, were lower. Nevertheless, the iron content in fat-free liver tissue of affected birds in the category >200–250 g fat/kg DM liver tissue (Fig. 2) was significantly higher than in livers of dead animals with less than 200 grammes of fat and those with more than 400 grammes of fat.

## Discussion

Hepatic lipidosis in turkeys is a disease that is accompanied by a massive fat accumulation in liver tissue as well as being the cause of high animal losses [1, 2, 12, 13]. The disease was described in Canada for the first time [1]. Both, a metabolic or an infectious genesis is suspected, but the exact cause of the disease has not yet been verified [1, 2, 12], also not in other birds [14]. To date, there are only very few studies which describe the functional characteristics of the turkey liver [15]. Livers of turkeys that died from hepatic lipidosis have not yet been analysed systematically for fat content, fatty acid profile and iron content by classical chemical methods. The comparative analysis of the fatty acid pattern, differences and shifts in the composition of the fatty acids can help to understand the pathogenesis, especially in a comparative consideration to other species [6, 11, 16–18]. In addition, the concentration of specific fatty acids in the liver is a good biomarker for assessing non-alcoholic fatty liver disease (NAFLD) in animal models [19].

In the current study, an increase in liver fat-% was seen (2.8 fold; 2.5 fold concerning the sum of fatty acids). An accumulation of most fatty acids in the liver of animals that died from hepatic lipidosis was detected. Only the contents of arachidonic acid, eicosapentaenoic acid and docosahexaenoic acid were significantly lower.

Palmitic acid is considered to be a toxic fatty acid for the liver [6, 20, 21]. In comparison to humans [6], the content of this fatty acid was numerically slightly higher in healthy turkeys (8.26 ± 2.57 g/kg liver tissue in turkeys versus 5.45 ± 0.670 g/kg liver tissue in humans). In humans [6], the contents in simple steatosis and steatohepatitis are significantly higher. That was also the case in the present study, albeit in livers of the highest fat category, values from humans were not reached (47.6 ± 4.69 g/kg at >450 g fat/kg liver tissue versus 52.8 ± 8.03 in SS and 92.8 ± 21.0 g/kg in NASH liver tissue for C16:0). The situation is similar for oleic acid, the unsaturated product of conversion of palmitic acid [6]. In palmitoleic acid in the highest fat category, however, the concentrations of the fatty acids are comparable to those of people with steatohepatitis (10.5 ± 3.29 g/kg liver versus 10.9 ± 2.35 g/kg liver tissue in people with NASH).

For birds, liver is the main site of fatty acid synthesis [22, 23]. Storage of large quantities of fat triglycerides in the liver as an energy source is a biological mechanism in migratory birds [24, 25]. This mechanism is analogous to human medicine. Like migratory birds, humans with an excessive calorie consumption also deposit fat in the liver [24]. Unlike poultry, fatty liver in humans is less adaptable [24]. Nevertheless, it makes sense to take a comparative closer look at the two phenomena. The earliest stage in the disease complex called nonalcoholic fatty liver disease (NAFLD) is hepatic steatosis [19]. In humans, steatosis is defined as a hepatic triglyceride level exceeding >55.0 mg per g of liver [24]. In this study, livers of control animals had a fatty acid content of 35.3 ± 10.3 mg per g of liver tissue.

Fatty liver in birds occurs whenever the increase in lipogenesis exceeds the capacity for synthesis and secretion of lipoproteins [15]. Physiologically, this occurs naturally under estrogen dominance as a so- called fatty liver hemorrhagic syndrome in laying hens. In turkeys, hepatic steatosis is different and a condition that particularly favours development of hepatic lipidosis has yet to be determined [15]. In humans, the increased supply of free fatty acids to the liver from the diet, from adipose tissue, and through increased *de novo* lipogenesis all serve to promote hepatic steatosis [24]. About 59% of hepatic fat is derived from circulating free fatty acids, lower percentages coming from *de novo* lipogenesis (26%) and the diet (15%) [26]. In chickens, the liver is the major site of fatty acid synthesis [22]. During fasting, liver lipid is mobilized rapidly [22]. Fasting reduces lipogenesis and increases lipolysis [22]. In voles (Arvicolinae) fasted for 18 h, increase in liver fat-% was statistically significant (2-fold) [18].

As described for nonalcoholic steatohepatitis in humans [6], it could also be verified in this study that the ratio of C18:0/C16:0 falls and the ratio of C16:1n7/C16:0 rises. The former is correlated with inflammation and the ballooning of hepatocytes in the liver tissue, the latter with an inflammation of the liver tissue in general [6]. For a detailed assessment of liver disease, the so-called metabonomic biomarker is used as a parameter [19]. Thus, lower concentrations of arachidonic acid, eicosapentaenoic acid and docosahexaenoic acid are good markers. In the present study, these acids were found in lower concentrations in suspicious liver tissue, too.

The iron content in the liver was analysed since infectious and inflammatory diseases may lead to an accumulation of iron in the liver in specific cases [27–30].

On the one hand, significantly higher iron contents in the liver tissue were found in turkeys died from hepatic lipidosis in comparison to healthy animals. On the other hand, it was possible to demonstrate that in animals that had died from hepatic lipidosis, the sum of the fatty acids in the liver showed a negative correlation to the iron content in the liver (PCC: −0.67; *p* < 0.01). In principle, this means that the accumulation of fat in the liver under the condition of hepatic lipidosis is associated with a higher concentration of iron. However, with increasing fat content, the iron concentrations decreased. In healthy poultry, liver contains concentrations of more than 60 mg/kg iron (equivalent to about 220 mg/kg DM) in fresh tissue [31]. The control livers of healthy animals therefore had normal iron contents in this study, which should by no means be described as high. Only levels of more than 300 mg/kg iron in liver tissue may be referred to as high [31]. Tissue injury due to excess iron eventually leads to organ dysfunction [32, 33]. In general, liver damage (hepatic fibrosis, cirrhosis) occurs when liver iron concentration increases to more than 10-times the normal level [34]. This means that there was indeed a significant increase in liver iron levels. However, it cannot be assumed that there was liver damage induced only by iron accumulation in liver tissue in this study.

Slight differences in the iron content of the liver tissue can be caused by a different residual blood content alone. However, there are no studies on the residual blood content in turkey livers. From studies in rats [35], differences in residual blood contents in the liver tissue of 0.11 g/cm^3^ are known. With an estimated iron content of 0.44 mg/g blood ([36]; values from pigs) this would corresponds to a possible difference in the iron content in liver tissue (with or without bleeding) of 48.4 mg/kg liver.

It is still unknown as to what stimulated the accumulation of iron in the liver. Hepcidin is the main regulator of iron homeostasis in vertebrates [28, 37–39]. The liver plays a central role in regulating iron homeostasis because the liver is the main producer of hepcidin as well as being the main iron depot in the body [7, 38, 39]. It is known that both, cytokines and hepcidin, force the iron retention in the spleen, liver and bone marrow macrophages [30, 40]. Normally, the synthesis is induced by systemic iron levels and by inflammatory stimuli [34, 37]. The liver responds to inflammatory signals originating extrahepatically by increasing the hepcidin level [38]. Infections or stimuli that are likely to induce liver hepcidin expression, reduce serum iron and increase iron accumulation in reticuloendothelial cells [38, 41].

In three cases of hepatic lipidosis among turkey breeder flocks, virus particles similar to parvo- or picornavirus were detected via electron microscopy [2]. In addition, picornavirus RNA was found in the livers of one turkey flock with PCR techniques [2]. In a case report, which refers to the data of this previous study on hepatic lipidosis, antibodies against Avian Encephalomyelitis Virus and genome fractions of Turkey Viral Hepatitis Virus were found [4]. In fact, it cannot be ruled out that a specific infectious agent causes the iron accumulation in the liver.

Iron accumulation also occurs in other noninfectious disorders of the liver [7, 39]. The expression of hepcidin, for example, is also stimulated by adipokines [40, 42]. Hepcidin levels were significantly higher in obese children with NAFLD than in those without NAFLD [43]. According to the present study, therefore, changes in the hepcidin regulation would be possible but should be the focus of future investigations.

## Conclusions

In summary, there are two aspects that have to be underlined: On the one hand, the development of a fatal fatty liver disease, on the other hand the changes in iron concentrations in the liver. In the genesis of NASH in humans “two hits” arise [44, 45]. In a first stage there is a hepatic steatosis. In a second stage, proinflammatory cytokines lead to oxidative stress and liver damage [44]. It can be assumed that diet and feeding cause a certain predisposition to a fatty liver. As an attendant characteristic of an infection, there is a cytokine mediated reduced feed intake [46]. This may foster the development of fatty liver disease in turkeys, too. By infection-related release of inflammatory mediators and a derailment of iron metabolism further damage of the liver tissue occurs ultimately leading to the death of affected animals. This could be also an explanation for the high animal losses in the case of hepatic lipidosis in turkeys.

In general, the turkey could thus serve as a good animal model for certain metabolic and infections related metabolic problems in human medicine because the animal is sensitive to various triggers and shows typical mechanisms of liver disease.

## Abbreviations

DM: Dry matter
fl: Fatty acid class
NAFLD: Nonalcoholic fatty liver disease
NASH: Nonalcoholic steatohepatitis
SS: Simple steatosis
